# Silver Nanoparticles Help Plants Grow, Alleviate Stresses, and Fight Against Pathogens

**DOI:** 10.3390/plants14030428

**Published:** 2025-02-01

**Authors:** Francisco Javier Alfosea-Simón, Lorenzo Burgos, Nuria Alburquerque

**Affiliations:** Group of Fruit Tree Biotechnology, Department of Plant Breeding, CEBAS-CSIC, University Campus at Espinardo, Bldg. 25, 30100 Murcia, Spain; fjalfosea@cebas.csic.es (F.J.A.-S.); burgos@cebas.csic.es (L.B.)

**Keywords:** nanobiotechnology, sustainable agriculture, nanofertilizer, nanopesticide, abiotic stress, biotic stress

## Abstract

The use of silver nanoparticles (AgNPs) has gained importance in agriculture in recent years thanks to their unique characteristics, including their antimicrobial capacity and their ability to promote plant growth. Due to these attributes, AgNPs are considered a promising solution for the future of agriculture, offering significant potential to address the challenges the sector confronts currently. However, it is important to adjust the application conditions, depending on the target and the crop used, to improve AgNP treatment efficiency. This review compiles recent advances in the use of AgNPs for crop production, both in and ex vitro. AgNPs promote growth and alleviate biotic and abiotic stresses through different ex vitro application methods. They are also efficiently used in vitro to improve plant culture and pathogen elimination. In addition, the safety and toxicity associated with their use are discussed. AgNPs are a novel tool with great potential for the agricultural sector, but it is still necessary to continue researching the mechanisms of AgNP action in order to optimize their application in each specific case.

## 1. Introduction

Nanotechnology is the science dedicated to the study of matter at the nanometer scale (1–100 nm). It is considered a crucial discipline for long-term progress in various areas and has gained more relevance in recent years. Despite its numerous industrial applications, its implementation in agriculture is not so generalized. However, the study of these new tools in this field could be fundamental to ensure food security in a long-term perspective [1] because, in the current context of climate change, in which the world is heading towards warmer and more extreme conditions, combined with the increase in world population and the lack of natural resources, making improvements in food quality and production is an important priority for society [2]. There is ample scientific evidence that demonstrates the significant risks that conventional pesticides present to human health and the environment [3,4]; therefore, the correct use of new tools, such as nanopesticides, could be of great value for agriculture, as they can be more effective than conventional alternatives [5]. On the other hand, the use of nanoherbicides may be a promising strategy against the current problem of weed control, since in some cases, they have generated resistance to the current ones. Nanoherbicides can help in this situation because they can be applied at low doses and with a controlled release [6]. There are more examples where nanotechnology could be used in agriculture, including processes like packaging and commercialization [7].

This discipline is based on the premise that the properties of materials at the nanoscale present different behavior and properties than those observed in their conventional form. For example, changes are observed in the optical, electrical, interfacial, and tensional properties, in addition to presenting greater reactivity and catalytic activity, which allows for obtaining new functional materials and nanoparticles (NPs), with a wide range of novel uses [8]. Nanoparticles have these distinctive characteristics, mainly due to their small size and large surface area [9].

NPs can be classified according to different criteria, such as their origin, dimensionality, shape [10], size [11], or chemical characteristics [12]. For classification according to their origin, a distinction can be made between NPs of the following types: natural, incidental (ultrafine particles obtained as unintentional by-products of controlled processes), and engineered; in terms of dimensionality, NPs are categorized into four types: zero-dimensional, one-dimensional, two-dimensional, and three-dimensional; according to their shape, a wide variety of forms exist, including spherical, triangular, cubic, hexagonal, oval, prism, rod, tube, helical, etc. [10]; there are also complex forms for specific functions, such as nanocages, and even much more complicated forms that use DNA as a base structure [13]; and finally, according to the criteria of chemical characteristics, the following groups are recognized: carbon-based NPs, metal NPs, ceramic NPs, lipid-based NPs, semiconductor NPs, and polymeric NPs [12].

The use of metallic nanoparticles (MeNPs) has different applications in the medical field thanks to their antimicrobial [14] and antiviral properties [15]; they can even be used as a treatment for cancer [16]. In agriculture, MeNPs have also been used as nano-biofertilizers to improve plants’ adaptation to abiotic stresses in a climate change scenario [17,18]. These versatile properties have generated great interest in the use of this group of NPs in agriculture, among which silver nanoparticles (AgNPs) are particularly interesting, thanks to their remarkable antimicrobial and especially antiviral capabilities [19,20].

The purpose of this review is to provide an updated and detailed view of AgNPs and their use in agriculture and plant species, expanding on their main applications to achieve plants that respond better to biotic and abiotic stresses; offering a global perspective on this field that can serve as a solid basis for future research. A total of 130 scientific publications have been reviewed for this purpose.

## 2. Synthesis Methods

The synthesis of AgNPs dates back more than a century, with advances such as the creation of citrate-stabilized silver colloids (with sizes of 7 to 9 nm) reported almost 120 years ago, and the development of “Collargol” in 1897, a silver nanoparticle used for medicinal purposes [12].

**Figure 1:**
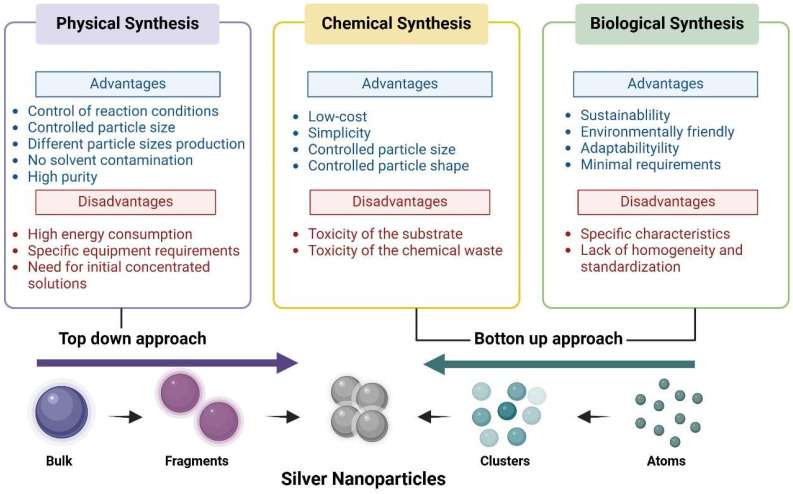
Types of silver nanoparticle synthesis methods. This figure was created using BioRender (https://biorender.com/).

AgNPs can be synthesized using various methods broadly categorized into physical, chemical, and biological or green methods (Figure 1).

Physical methods for synthesizing AgNPs, such as electricity, light energy, microwave plasma, and evaporation–condensation, involve decomposing synthetic media to produce reducing substances (e.g., ethanol and ethylene glycol) that react with silver ions to form AgNPs [21]. These methods allow precise control of reaction conditions to produce AgNPs of varying particle sizes, with the advantage of the absence of solvent contamination and high purity. However, they have drawbacks, including high energy consumption and the need for concentrated solutions [21]. The process heavily relies on the choice of the synthesis medium, which is typically an organic solvent or water. Distilled or deionized water is the most commonly used medium due to its low cost, safety, and high heat capacity [22].

Reduction, the most common chemical method of AgNP synthesis, uses different compounds (citrate, ascorbic acid, sodium borohydride, or block copolymers) to trans-form silver ions (Ag+) into nanoparticles. The first step for AgNP formation is the production of neutral silver atoms to form clusters which lead to the production of AgNPs with different sizes and shapes [23]. This approach requires a metal precursor, a reductant, and stabilizing agents, such as polyvinylpyrrolidone (PVP), to ensure the stability of chemically synthesized colloids, all of which are present in the AgNP solution. Therefore, the substrate and chemical waste can pose risks to human health [24]. Despite these limitations, the method remains widely used due to its low cost, simplicity, and overall convenience in producing nanoparticles with controlled sizes and shapes [25].

Biological or green synthesis methods use natural compounds as reducing and stabilizing agents instead of harmful substances, resulting in a sustainable and environmentally friendly alternative to chemical processes [26,27]. Plant extracts with phytochemical compounds (flavonoids, terpenoids, glycosides, alkaloids, and phenolics) serve as reducing agents to synthesize AgNPs from metal salts [27,28]. Different studies have reported that the properties of synthesized AgNPs are influenced by the specific characteristics of the plant or the kind of extract [23]. Also, biological methods employ microorganisms, such as bacteria [29] or fungi [30], to synthesize AgNPs. The microbiological synthesis of AgNPs involves two main pathways. In one pathway, microorganisms secrete reductase enzymes and proteins, which reduce silver ions and stabilize AgNPs on their surface. In the other, reductase released by microorganisms reduces silver ions after the microorganisms are removed, producing smaller particles. Due to their adaptability, rapid growth, and minimal requirements, microorganisms are widely studied for AgNP synthesis [23].

Nowadays, physical methods use a top-down approach for the synthesis of NPs; meanwhile, chemical and biological methods apply a bottom-up approach (Figure 1). In the top-down approach, suitable bulk material is split into fine particles by size reduction with different techniques [23]. In the bottom-up approach, through chemical or biological methods, nanoparticles can be synthesized through the phenomenon of self-assembly of atoms into new nuclei that grow into nanoscale particles [12,23].

## 3. Applications

### 3.1. Nanofertilization

**Figure 2:**
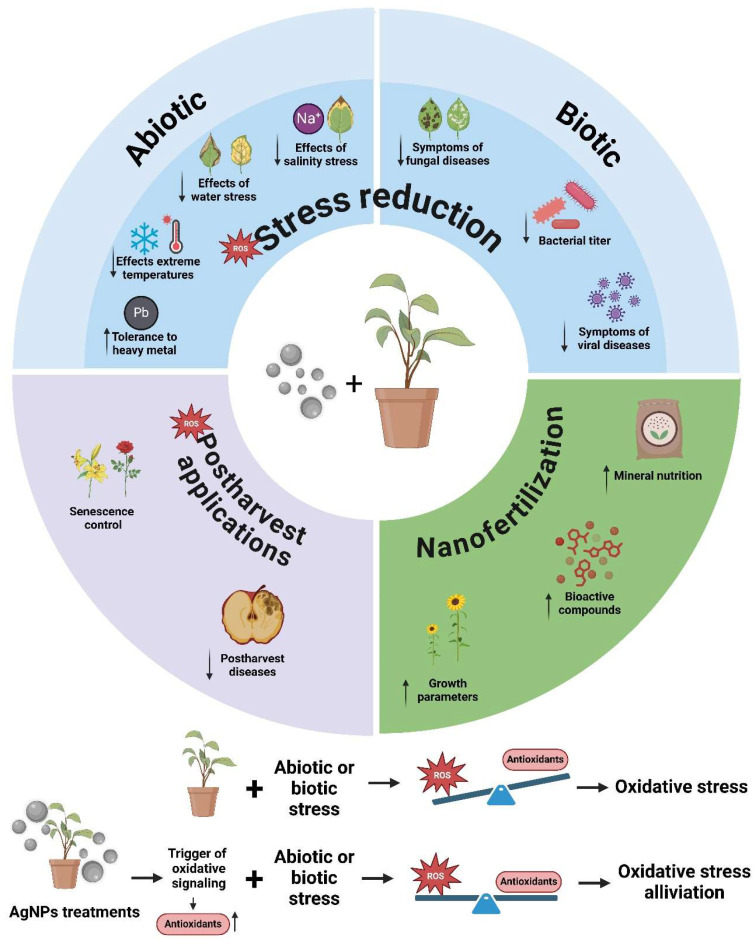
Overview of ex vitro applications of silver nanoparticles (AgNPs) in agriculture (circular diagram) and possible mechanism of AgNP action. This figure was created using BioRender (https://biorender.com/).

The use of silver nanoparticles has shown positive effects on the growth and development of multiple crops when used under controlled conditions, with different application methods (Figure 2) [31]. The authors Berahmand et al. [32] obtained yield improvements in maize (*Zea mays* L.) by applying AgNPs in irrigation water (40 g ha^−1^) along with a magnetic field treatment. On the other hand, its foliar application increased root nodule production in cowpea (*Vigna sinensis* L.) plants and shoot length in mustard (*Brassica juncea* L.), at an optimal dose of 50 mg L^−1^, and promoted vegetative development in cowpea plants when foliar application of 75 mg L^−1^ of this nanoparticle was carried out [33]. Exogenous application of 60 mg L^−1^ of AgNPs to sunflower (*Helianthus annuus* L.) plants, both to seeds and as a foliar spray (and their combined treatment), not only positively affected plant growth, but also improved the contents of biochemical compounds, antioxidants, fatty acids, and secondary metabolites [34]. In bell pepper (*Capsicum annuum* L.) plants irrigated with untreated wastewater, it has been observed that its foliar application of 80 mg L^−1^ improved mineral nutrition in several tissues and improved growth, in addition to improving the nutritional properties of the fruit, such as crude fibers, proteins, and carbohydrates. This makes them a suitable, cost-effective, and environmentally friendly strategy when using low-quality irrigation water [35]. Its use on fenugreek (*Trigonella foenum-graecum* L.) seedlings (1 mg L^−1^ of AgNPs was added to each seedling and they were kept for 5 days) resulted in a significant increase in growth parameters (number of leaves, root length, shoot length, and fresh weight), with respect to the control, not treated with AgNPs, in addition to an enhancement in the biosynthesis of the compound diosgenin, which plays an important role in disease resistance and other growth properties of the plant and is known for its medicinal properties [36]. This work demonstrates the effective use of AgNPs as nanoelicitors.

The exogenous application of the commercial silver nanoparticle formulation Argovit^TM^ (20 mg L^−1^), in harvested carrots (*Daucus carota* L.), acted as a controlled abiotic stress, which increases the production of high-value bioactive compounds such as chlorogenic acid, 3-O-caffeoylquinic acid, 5′-caffeoylquinic acid, and ferulic acid, which present anti-inflammatory, anticarcinogenic, antidiabetic, hepatoprotective, cardioprotective, and neuroprotective actions [37]. Its post-harvest application can also be used to prolong the longevity and increase the quality of cut flowers like Lily (*Lilium* spp.), Lisianthus (*Eustoma* spp.), Rose (*Rosa* spp.), or Carnation (*Dianthus caryophyllus* L.), among other species, (with different optimal doses), as it has been seen that AgNPs are able to kill the bacteria present in the water of the vases and the ends of the stems. In addition to that, AgNPs can move through the xylem to flowers where they reduce ethylene production by suppressing the genes involved. This results in AgNP-treated cut flowers having higher relative water content, higher fresh weight, and higher antioxidant activity [38].

Additionally, a positive interaction between AgNPs and plant growth-promoting rhizobacteria (PGPR) has been observed, which may help to enhance their beneficial properties [39]. However, this beneficial effect when applying both treatments combined was not seen in maize seedlings. The individual treatments with *Bacillus cereus* (LPR2 strain) or AgNPs (synthesized from marigold (*Tagetes erecta* L.) leaf and flower extract) increased germination (87.5%) while the combining treatment LPR2 + AgNPs obtained a germination of 75%. The same occurs with the growth of root and shoot, as it was higher in the treatment with LPR2, followed by the treatment with AgNPs and the combination of both [40].

**Figure 3:**
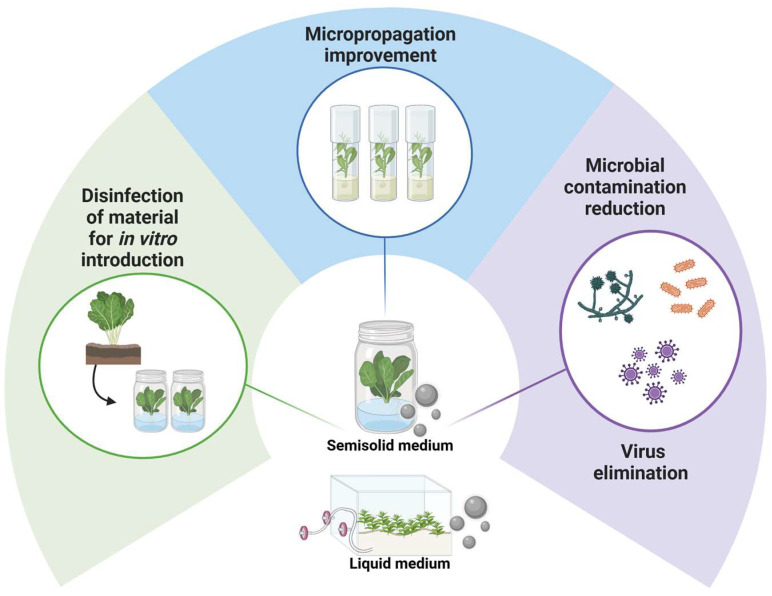
Applications of silver nanoparticles (AgNPs) for in vitro plant cultivation. This figure was created using BioRender (https://biorender.com/).

In addition to their exogenous or ex vitro application, there are a large number of studies describing the use of AgNPs as a useful tool for the in vitro culture of different plant species (Figure 3). The addition of these nanoparticles to the culture medium, either semi-solid or liquid, increased the propagation and biomass (6 mg L^−1^ enhanced shoot multiplication from 1.29 ± 0.14 to 25.17 ± 0.45 in night-blooming jasmine (*Nyctanthes arbor-tristis* L.) plants) [41]. Also, increases in biomass and non-enzymatic antioxidants (phenol, tannin, and flavonoid content), total protein content, superoxide dismutase (SOD), and bioactive compounds like gallic acid, tannic acid, coumarin, hesperidin, rutin, quercetin, and ferruginol were found in African juniper (*Juniperus procera* Hochst. ex Endl.) plants grown in a semi-solid medium with 5, 20, and 50 mg L^−1^ of AgNPs [42]. The activity of the antioxidant enzymes ascorbate peroxidase (APX) and SOD was improved in in vitro plants of lavender (*Lavandula angustifolia* Mill.) after AgNP treatments of 2 and 5 mg L^−1^ [43]. A hormetic effect was found in stevia shoots (*Stevia rebaudiana* Bertoni) grown in a liquid medium when a commercial formulation of Argovit^TM^ was added, which stimulated growth and chlorophyll content at lower studied concentrations (50 mg L^−1^) [44].

In sugarcane (*Saccharum* spp.), similar results were obtained with the same dose of Argovit^TM^, which optimally stimulated growth. The presence of AgNPs in the medium induced changes in macro- and micronutrient content, induced reactive oxygen species (ROS) production, and increased total phenolic content, dependent on the dose used [45]. Likewise, the same dose of this product added to the in vitro liquid culture medium in gerbera (*Gerbera jamesonii* Adlam.) flowers was able to increase their life once cut to 9.8 days compared to the untreated control (8.2 days) [46].

There are a large number of examples of the use of AgNPs in in vitro culture in both semi-solid and liquid media [47]. However, the type of medium used can influence the effectiveness of AgNPs and may even require modifications in their composition, as shown in the work performed on apricot (*Prunus armeniaca* L.) by Pérez-Caselles et al. [48]. The shoots grown in a semi-solid medium were not able to take up the silver nanoparticles added to the medium because the agar matrix caused the immobilization of the AgNPs. Therefore, to ensure their assimilation, it was necessary to use a liquid medium and Temporary Immersion Systems (TISs), in addition to modifying the medium (elimination of chlorides to avoid precipitation of silver salts). Under these conditions, an increase in the proliferation and biomass of apricot shoots was achieved with an optimal dose of 75 mg L^−1^ of the commercial formulation of AgNP Argovit^TM^-7 [49].

The benefits of AgNPs in in vitro culture are not limited to enhancing growth, proliferation, and compound synthesis, as demonstrated by Andújar et al. [50] who describe that, thanks to the antimicrobial effect of AgNPs, they were able to reduce microbial contamination when establishing guava plants (*Psidium friedrichsthalianum* (O. Berg) Nied.) in in vitro culture (a frequent and very important problem) by immersing the shoots in Argovit^TM^ solutions before transferring them to a semi-solid culture medium; they also observed improvements in leaf area and multiplication rate. This same commercial product was used by Spinoso-Castillo et al. [51] with similar results against microbial contamination in vanilla (*Vanilla planifolia* Andrews). These authors were able to reduce the contaminations produced by bacteria by adding doses of 50, 100, and 200 mg L^−1^ of Argovit^TM^ to the liquid medium. In addition, an improvement in crop micropropagation was observed at the optimum dose of 50 mg L^−1^, as well as an increase in ROS production, total phenolic content, antioxidant capacity, lipid peroxidation, and changes in macro- and micronutrient content. Other examples where AgNPs have been used to disinfect plant material during in vitro establishment include works with strawberry (*Fragaria x ananassa* Duchesne ex Rozier) [52], rubber (*Hevea brasiliensis* (Willd. ex A. Juss.) Müll. Arg.) [53], and an almond x peach hybrid [54]

All this shows that the interaction between plants and silver nanoparticles is very complex, and it is necessary to optimize the concentration and type of application to promote the optimal development of each crop, without inducing toxic effects. It is also necessary to study the mechanisms responsible for the different responses of each plant species, in order to be able to apply them successfully in a commercial way.

### 3.2. Abiotic Stress Reduction

In the current context of climate change, the incidence of multiple abiotic stresses associated with it causes a decrease in agricultural productivity. Water and soil salinity, extreme temperatures, water stress, and the presence of heavy metals in crop fields have been reported in numerous studies as causes of a severe reduction in yields. Abiotic stresses are known to provoke numerous reactions that negatively impact plant cellular processes, as well as produce oxidative and osmotic effects, which restrict plant growth and productivity [55]. In the face of this situation, the use of AgNPs, thanks to their ability to strengthen the antioxidant capacity and to alleviate oxidative stress (Figure 2, possible mechanism of AgNPs), has been described as an appropriate strategy to overcome abiotic stress in different plant species, with great potential to address this problem [56].

#### 3.2.1. Salinity

Soil and water salinity, along with associated problems, is one of the major abiotic constraints to global food production and is especially critical in semi-arid and arid regions [57]. This problem is aggravated by the advance of the climate crisis, which intensifies soil salinization processes and further limits the availability of adequate water resources for agriculture [58]. Salt stress causes alterations in plant morphology and physiology, as well as in their biochemical and molecular processes, affecting their growth and, consequently, crop yields [59]. Salts present in water or soil can inhibit plant growth for two reasons. First, salt reduces the plant’s ability to absorb water, leading to a decrease in growth rate. Second, if excessive amounts of salt enter the plant through the transpiration stream, they can cause cell damage and ionic imbalances [60].

Numerous examples exist in which AgNPs have been used as a strategy to counteract the effects of salinity stress in a large number of crops, highlighting priming with AgNPs as a very effective strategy to enhance salt tolerance in numerous crops [61], such as wheat (*Triticum aestivum*) [62,63], thyme (*Thymus vulgaris* L. and *Thymus daenensis* Čelak) [64], pearl millet (*Pennisetum glaucum* L.) [65], and cimbru (*Satureja hortensis* L.) [66].

Yan et al. [67] studied in depth the effect of priming with AgNPs in rice seedlings (*Oryza sativa* L.) in addition to finding faster germination and higher growth in the primed seeds; thanks to their metabolomic and transcriptomic study, they observed an increase in key metabolites such as salicylic acid, niacinamide, and glycerol-3-phosphate, which are essential in the stress response. Metabolic pathway analysis showed activation of pathways related to hormone signaling, glutathione metabolism, flavone and flavonol biosynthesis, the mitogen-activated protein kinase signaling pathway, and plant–pathogen interaction. These changes suggest that treatment with AgNPs may activate “stress memory” responses, providing long-lasting protection against abiotic and biotic stresses.

However, the application of AgNPs against salt stress is not limited to the priming technique. For example, in onion (*Allium cepa* L.) seedlings, both individual and combination treatments with AgNPs (5 mg L^−1^ applied to rhizosphere soil for 5 days) and PGPRs (*Bacillus pumilus* and *Pseudomonas moraviensis*) increased soil moisture retention as well as chlorophyll and carotenoid contents, improved protein content in the bulbs, and improved bulb protein content when seedlings were treated with 50 mM NaCl for 7 days [68]. Khalofah et al. [69] evaluated the combined effect of the bacterium *Comamonas testosteroni* and foliar applications of AgNPs on the growth of linseed (*Linum usitatissimum* L.) subjected to salt stress (NaCl concentrations: 0, 25, 50, 100 mM). Beneficial effects were found both in the separate application of these strategies and in their combined use. The treatments increased photosynthetic pigment production and raised soluble sugars, proline, and protein levels; the combination of both treatments further enhanced enzymatic and non-enzymatic antioxidants while reducing H_2_O_2_ and malondialdehyde, effectively used as a biofertilizer in saline soils. Foliar application has also achieved good results in maize plants since it was able to improve the detrimental impacts produced by a high salt concentration (80 mM), such as the reduction in agronomic attributes, photosynthetic pigments, osmolytes, and antioxidant enzymes, through the foliar application of indole acetic acid (IAA) and citrate-capped silver nanoparticles, both individually and in combination [70].

#### 3.2.2. Water Stress

Drought is an abiotic stress that negatively affects plant growth and yield by interfering with the flow of water in plants. Water scarcity affects key processes such as water transport, osmoregulation, and cell expansion in plants, which can compromise food security [56]. Similar to salt stress, there are a large number of studies that successfully utilize AgNPs to mitigate the effects of drought stress.

An in vitro study on boysenberry (*Rubus ursinus* Cham. & Schltdl.) plants showed that the addition of AgNPs to the culture medium mitigated the adverse effects of drought stress simulated with Polyethylene Glycol, observing an increase in growth parameters and antioxidant activity, especially for SOD and catalase (CAT) enzymes, and a decrease in malondialdehyde (MDA) [71]. These positive effects against drought stress have also occurred under ex vitro conditions in numerous crops, for example, in eggplant (*Solanum melongena* (Mill.) Dunal) seedlings [72], lentil (*Lens culinaris* Medik.) seeds [73], lettuce (*Lactuca sativa* L.) [74], tomato [75] and wheat [76,77].

On the other hand, there are not so many studies in which AgNPs are used to alleviate water stress caused by excess water (flood). Rezvani et al. [78] in a field trial managed to improve the effects produced by flood stress (a type of stress frequent in autumn due to rainfall) in the saffron (*Crocus sativus* L.) crop by immersing the corms in solutions with different concentrations of AgNPs. Plants subjected to this water stress for 10 days were affected in their growth, reducing the number of roots and their length, as well as the fresh and dry weight of both roots and leaves. Under these conditions, AgNP treatments compensated for the negative effects of abiotic stress. Specifically, the 40 and 80 mg L^−1^ doses increased the root number, while the 40 mg L^−1^ concentration increased the root length and the 80 mg L^−1^ dose increased the leaf dry weight. This response to flooding stress in the presence of AgNPs could be understood thanks to the proteomic and transcriptomic study performed by Mustafa et al. [79] on soybean (*Glycine max* (L.) Merr.) plants. In this case, AgNP treatments also helped to improve plant growth under stress conditions. These authors were able to identify differences in the expression of numerous root proteins, such as glyoxalase II 3 and other fermentation-related proteins, which increased with flooding, but decreased when plants were treated with AgNPs. At the transcriptomic level, alcohol dehydrogenase 1 (ADH1) and pyruvate decarboxylase 2 (PDC2) genes were expressed more under stress conditions, but AgNP treatments reduced their expression.

#### 3.2.3. Temperatures

The use of AgNPs has also proven to be an effective option to increase plant tolerance to low temperatures. In a study conducted on green beans (*Phaseolus vulgaris*), the application of low concentrations of AgNPs (0.25, 1.25 mg L^−1^) in the seedbed showed a positive impact on germination (faster and more uniform), both at conventional temperatures (25/20 °C day/night normal temperature) and at low temperatures (15/10 °C chill temperature); even improvements in plant height, fresh and dry weight, and photosynthesis were observed in plants grown in field conditions [80].

AgNPs have also shown good results against high-temperature stress. In wheat plants, NP treatments improved the antioxidant defense systems during heat stress conditions (35–40 °C for 3 h/ day for 3 days applied to wheat plants at the trifoliate stage). In this study, an improvement in the levels of chlorophyll a and b, as well as in the total chlorophyll content, was observed, which favors photosynthetic efficiency. In the antioxidant system, the activities of key enzymes such as SOD, peroxidase (POX), catalase (CAT), APX, and glutathione peroxidase (GPX) were increased, protecting cells against oxidative damage [81].

#### 3.2.4. Heavy Metals

The widespread use of heavy metals (HMs) in different sectors such as agricultural, industrial, medical, and domestic has led to an increasing accumulation of HMs in the environment, causing a serious problem nowadays. In plants, heavy metals can alter metabolic processes essential for plant growth, causing symptoms such as root browning, chlorosis, growth delays, and even plant death [82].

Treatments with AgNPs have also proven to be an effective option against some HMs. In the study by Zhu et al. [83], treatments with AgNPs (15 and 30 mM) were used in combination with PGPR on barley (*Hordeum vulgare* L.) subjected to Cr stress. These treatments were able to mitigate the negative effects of this metal as they were able to increase plant growth, improve the photosynthetic apparatus, and enhance antioxidant enzyme and mineral uptake, as well as decrease organic acid exudation and oxidative stress indicators. Similarly, AgNP treatment has been shown to be effective against the toxic effects of Pb in mung bean (*Vigna radiata* (L.) R. Wilczek), as it helped to improve plant growth and physiology. A dose of 25 mg L^−1^ favored an increase in plant height, fresh biomass, and water use efficiency, as well as helped to reduce the negative effects of Pb, such as oxidative damage and suppression of photosynthetic activity [84].

According to Azeez et al. [85], their positive effect against HM toxicity can be explained by the action of two mechanisms. On the one hand, their ability to immobilize them reduces their absorption, and on the other hand, AgNPs have the capacity to reduce oxidative damage.

### 3.3. Biotic Stress Reduction

Since the beginning of agriculture, producers have been confronted with a large number of pests and diseases, being one of the main causes of crop damage, reducing the quality of harvests, and threatening the economic viability of agricultural farms worldwide [86]. Crops can be affected by a wide range of pathogens and pests, such as insects, mites, myriapods, nematodes, parasitic weeds, fungi, bacteria, and viruses [87]. As shown in Figure 2, AgNP treatments can trigger the oxidative signal and increase the antioxidant capacity of treated plants, so that when they are exposed to biotic stresses, oxidative damage is reduced. The use of AgNPs has proven to be a good strategy against fungi, bacteria, and viruses that affect plants, as explained below.

#### 3.3.1. Fungi

AgNPs have been successfully employed for treatments against many fungal diseases (Table 1). They have been shown to be able to effectively inhibit mycelial growth and spore germination in vitro of four pathogens responsible for kiwifruit (*Actinidia deliciosa* (A.Chev.) C.F. Liang & A.R. Ferguson) rot: *Alternaria alternata*, *Pestalotiopsis microspora*, *Diaporthe actinidiae*, and *Botryosphaeria dothidea*, with an optimum dose of 75 mg L^−1^. This same dose applied directly to the fruit by spray also showed good capacity to reduce the symptoms, without leaving Ag residue in the pulp and peel [88]. AgNPs synthesized using black tea extract showed good antifungal capacity under in vitro tests against *Monilinia fructigena*, a fungus responsible for the Apple Fruit Brown Rot disease. A dose of 200 mg L^−1^ inhibited the radial growth of this fungus by 91%. In addition, the artificial co-inoculation of this pathogen with the same dose of AgNPs to the fruit reduced the diameter of the lesions (77.4%) and its fresh weight (84.4%). Therefore, AgNPs represent an interesting option to improve the post-harvest of fruits, in an ecological and economical way, since it has been demonstrated that they do not affect human health due to their null cytotoxicity against human HaCaT cells [89]. Al-Sheikh and Yehia [90] developed a composite of chitosan and AgNPs, with good antifungal activity under in vitro conditions, that is effective against the pathogens *Penicillium digitatum* and *Penicillium italicum* which are responsible for green and blue mold post-harvest diseases affecting fruits of the genus *Citrus*. The effect of the combined composite of chitosan and AgNPs exceeds chitosan alone.

Chemically synthesized AgNPs have been effective against the fungus *Bipolaris oryzae*, responsible for rice brown spot, both in in vitro and in vivo conditions. In the in vitro experiments, increasing concentrations of AgNPs (0, 1, 5, 10, 25, 50, 100, and 200 μmol L^−1^) reduced the mycelial growth speed, the mycelia diameter, and the germination rate of this fungus. In vivo greenhouse tests (foliar treatment of 5 μmol L^−1^) reduced the intensity of the disease, increased the amount of chlorophyll, and reduced the activity of SOD, CAT, POX, and APX enzymes, as a result of the decrease in cell damage. On the other hand, an increase in PAL enzyme activity was observed, which is linked to the activation of defense pathways [91]. AgNPs synthesized from *Ocimum sanctum* leaf extract were an effective treatment against *Alternaria porri* fungus both in vitro (maximum inhibition of 91.71% was obtained at a dose of 150 mg L^−1^) and in a field study in onion (maximum inhibition of 80.93% was obtained at a dose of 100 mg L^−1^ applied by foliar spray) [92].

Its antimicrobial effect has also been shown to be effective against fungi of the genus *Sordaria*, which is frequent in in vitro culture contamination. In stevia culture, concentrations of 50–100 mg L^−1^ were able to inhibit their growth up to 50%, while a concentration of 200 mg L^−1^ achieved much greater growth reductions [93].

#### 3.3.2. Bacteria

Despite its antibacterial properties, there are not many studies on the effect of AgNPs against bacteria that affect plants. However, the commercial formulation Argovit^TM^ has been successfully used in the cultivation of Mexican lime (*Citrus aurantifolia [Christm.] Swingle*) against the phytopathogenic bacterium *Candidatus Liberibacter asiaticus*, responsible for the Huanglongbing disease known as “yellow dragon disease”, which affects citrus species with devastating effects worldwide. After foliar administration of AgNPs or trunk injection, bacterial titer reductions of 80–90% were obtained. The concentration of metallic silver that produces 90% of bacterial titer reduction is 24 mg per tree which corresponds to 222 mmol of Ag per tree when applied by foliar spray, and the doses were up to 5 times lower when AgNPs were injected into the trunk. With this method, a 72–83% reduction in the number of bacteria was achieved [94].

Another study conducted for the bacterial disease blight (caused by the bacterium *Pseudomonas syringae* pv. *Syringae*) on different wheat cultivars found that AgNPs can improve the antioxidant properties of this crop both under normal conditions and in the presence of this pathogen, as its response is cultivar-dependent. This work emphasizes the potential of using AgNPs as an effective method for combatting plant diseases as an alternative to antibiotics, which would help reduce their adverse impacts on organisms and ecosystems [95]. Thus, this is an application of AgNPs with great potential for agriculture that needs to be studied for more specific cases.

#### 3.3.3. Virus

In plants, AgNPs have been used as a foliar spray to reduce or eliminate the symptoms of numerous viruses (Table 2). Their preventive application has shown good efficacy in reducing virus concentration and symptoms of *tomato mosaic virus* (ToMV) and *potato virus Y* (PVY) in tomato plants, at an optimal dose of 50 mg L^−1^, applied 7 days before infection. This Systemic Acquired Resistance (SAR) was evidenced by the increase in different parameters, regarding the healthy control, such as photosynthetic pigments, total soluble proteins, and the activities of peroxidase (POD) and polyphenol oxidase (PPO) enzymes; this has also been observed by electron micrographs showing that AgNPs are attached to the coat protein of both viruses [96].

Elbeshehy et al. [97] also obtained good results against *Bean Yellow Mosaic Virus* (BYMV) in plants of broadbean (*Vicia faba* L.) by applying AgNPs synthesized by extracellular agents from *Bacillus pumilus*, *B. persicus*, and *B. licheniformis*. These authors were able to reduce the symptoms caused by the virus when applying AgNPs at the same time as inoculation and with better results when applied 24 h later, since they were able to completely reduce the symptoms of the disease, with a dose of 100 mg L^−1^. In this work, the preventive application prior to inoculation (72 h) showed no effect on the reduction in virus concentration. El Gamal et al. [98] also studied this same virus in *Vicia faba* L. and obtained similar results when using the same foliar dose, but 48 h after infection. They also obtained good results in the preventive treatment, doubling the dose used (200 mg L^−1^) and advancing the treatment 24 h before infection, achieving complete inhibition of the disease. In addition, the application of AgNPs stimulated the expression of the defense gene PR-1 and induced the production of defense-related oxidizing enzymes.

The application of AgNPs synthesized by spore crystal mixture of *Bacillus thuringiensis* achieved the total suppression of symptoms of *Sunhemp Rosette Virus* (SHRV) in guar bean (*Cyamopsis tetragonoloba* L.) plants when applied foliarly at the time of inoculation, with a dose of 50 mg L^−1^ [99]. AgNPs’ curative application (24 h after infection) was effective against *tomato spotted wilt virus* (TSWV) in melde plants (*Chenopodium amaranticolor* Coste & Reyn.). At an optimum dose of 200 mg L^−1^, an inhibition percentage of 90.4% was achieved [100].

A dose of AgNPs of 50 mg L^−1^ applied 3 days after infection was also effective in preventing the development of *banana bunchy top virus* (BBTV) symptoms in banana plants (*Musa × paradisiaca* L.), with an infection rate of 36%. This dose of AgNPs increased dry weight and leaf area compared to untreated infected plants and produced significant changes in the concentration of carotenoids in infected plants and an increase in phenols, proline, and the antioxidant enzymes POX and PPO, in addition to presenting low genotoxicity. However, a dose of 60 mg L^−1^ was phytotoxic [101].

Under greenhouse conditions, foliar application of 0.1 mg L^−1^ of AgNPs biosynthesized using the aqueous extract of *Ocimum basilicum* promoted growth in pumpkin plants (*Cucurbita pepo* L.) and delayed the development of *cucumber mosaic virus* (CMV) symptoms when applied 24 h before and after infection, reducing CMV accumulation levels by 92% and 86%, respectively. There was also a significant increase in total soluble carbohydrates, free radical scavenging activity, antioxidant enzymes (PPO, SOD, and POX), total phenolic and flavonoid content, and the expression levels of pathogenesis-related genes (PR-1 and PR-5) and polyphenolic pathway genes (HCT and CHI) [102].

The use of AgNPs against plant viruses is not limited to foliar application in ex vitro plants. The commercial formulation of AgNPs, using Argovit^TM^-7, has been used for in vitro culture to propagate apricot plants free of *Plum Pox Virus* (PPV). In this work, AgNPs were added to the liquid culture medium and after two four-week culture cycles and meristem rescue, a very high PPV elimination efficiency was achieved (75%), using a dose of 75 mg L^−1^ [20]. This makes them an innovative tool for in vitro plant sanitation, which could be used in conjunction with other more common techniques such as thermotherapy, chemotherapy, cryotherapy, or electrotherapy [103].

## 4. Toxicity

Despite all the applications and benefits of AgNPs indicated in this review, it is important to know their possible toxic effects on crops, the environment, or human health. The growing relevance of AgNPs has raised concerns on this matter [104].

Regarding crops, an experiment conducted on *Nicotiana tabacum* L., whose roots were exposed to AgNPs and AgNO_3_ at equivalent concentrations, was able to demonstrate that AgNPs are less toxic than silver ions. Of all the concentrations studied (25, 50, 75, 100, or 500 μM), the nanoparticles did not produce, in any case, damage to root or leaf DNA (damage measured by the increase in DNA tail), unlike the AgNO_3_ treatments, which produced DNA damage in root tissue for all the concentrations studied and at the highest concentrations (100 and 500 μM) in leaf tissue. In addition, AgNPs did not cause an increase in oxidative stress parameters in roots or leaves in contrast to AgNO_3_ which induced oxidative stress in both tissues [105]. However, this statement may not be entirely accurate, since it is difficult to separate the action of AgNPs from silver ions. As observed in *Escherichia coli*, the toxicity of AgNPs is due to two mechanisms: a direct action through contact with bacterial cells and an indirect action derived from the release of Ag^+^ ions during the oxidative dissolution of the metal. This oxidation process, which persists over time, makes AgNPs potentially harmful agents if they are introduced into ecosystems [106]. It has also been demonstrated in experiments in *Escherichia coli* that a higher toxicity of AgNPs may be linked to a smaller particle size, since they have a higher specific surface area, which increases the area of contact with the bacteria, resulting in higher production of ROS [107]. This higher toxicity linked to smaller size observed in bacteria can occur similarly in plants [108] or animals [109].

The toxicity of AgNPs can also be related to other properties, such as its surface coating. Cvjetko et al. [110] studied the toxicity of three AgNPs with different coatings, citrate, polyvinylpyrrolidone (PVP), and cetyltrimethylammonium bromide (CTAB), on onion roots, observing that only AgNP-CTAB significantly inhibited root growth and cell proliferation at higher concentrations (75 and 100 μM) while AgNP–citrate and AgNP-PVP did not present that effect.

Moreover, the type of application used can influence the degree of toxicity, as observed in a comparative experiment conducted on lettuce plants, in which the effects of a root treatment with AgNPs (0.1, 0.5, and 1 mg L^−1^) and a foliar treatment (1, 10, and 50 mg L^−1^) were evaluated. These doses were established in order to be comparable in terms of effects on biomass (in both kinds of treatments, the higher dose produced fresh biomass decreased by around 40%). The results showed that root exposure induced a higher toxicity, which could be explained by the more efficient absorption and systemic transport of nanoparticles through the roots. In contrast, the foliar application of nanoparticles showed a more localized and limited uptake. In this work, it was seen that Ag is absorbed and transferred from the exposed parts to other areas, regardless of the mode of exposure [111]. These differences emphasize the importance of considering not only the concentrations applied or the type and properties of the AgNP used, but also the dynamics of absorption and transport that occur in plants.

In contrast, *Arabidopsis thaliana* (L.) Heynh. plants, regularly irrigated with AgNPs at a dose of 0.075 mg L^−1^, did not show remarkable morphological changes. This treatment prolonged vegetative development by two to three days and shortened flowering time by three to four days. However, germination rates of descendants decreased drastically over three generations, with this being the first time that this has been documented in plants [112]. AgNPs can negatively affect pollen performance, as demonstrated in an in vitro study on yellow poinciana (*Peltophorum pterocarpum [DC.] Backer* ex K. Heyne) pollen [113]. In this work, the effects of AgNPs and AgNO_3_ on pollen germination (%) and pollen tube length (μm) parameters were evaluated, finding significant reductions in these parameters for the range of concentrations studied (5 to 25 mg L^−1^), with the toxic effects being more pronounced with AgNPs compared to AgNO_3_. In addition, alterations in the structure of the exine (hard external cover of pollen grains) were observed by scanning electron microscopy, which could block germpores and interfere with pollen tube emergence. It has also been reported that AgNPs can alter the hormonal balance of plants, as indicated by Vinković et al. [114] in their work on bell pepper plants. Root treatments with AgNPs and AgNO_3_ at different concentrations (0.01, 0.05, 0.1, 0.5, and 1 mg L^−1^) affected plant development, decreasing its growth parameters and inducing a significant increase in cytokines in leaf tissue. All of the information above highlights the fact that there are multiple factors that can affect the interaction of AgNPs and plants, and their possible toxic effect, including factors of the AgNP itself and its usage mode as well as external factors [115], makes it difficult to generalize findings on this issue.

Although, in their use in agriculture, there are examples in which, with adequate application, no damage to crops is observed, it is essential to pay close attention to the possible release of AgNPs into ecosystems. Released AgNPs can undergo physical, chemical, and biological modifications, which can increase their toxicity in the environment [116] in both terrestrial [117] and aquatic ecosystems [118].

AgNPs can be contaminants when accumulated in soil due to their ability to induce oxidative stress. However, strategies such as the combination of silicon (Si) and PGPR treatments have been shown to be able to mitigate this toxicity, as demonstrated in a work on mustard [119] and rice [120]. In particular, the use of Si and PGPR, with or without IAA, has been able to restore normal plant growth and reduce oxidative stress, which could be a good solution to the problems of toxicity and contamination by AgNPs.

In the trophic chain, AgNPs can be accumulated by organisms at lower levels and transmitted to organisms at higher levels, as observed by Luo et al. [121]. In their work, they demonstrated that AgNPs accumulated in *Escherichia coli* could be passed by ingestion to the nematode Caenorhabditis elegans, and although AgNPs do not produce negative effects for *E. coli*, they did affect *C. elegans* specifically in terms of germ cell apoptosis, reproduction ability, and population size. Similarly, it has been observed that AgNPs accumulated in lettuce leaves from foliar treatments can be assimilated through ingestion by *Achatina fulica* snails [122], which highlights the ability of AgNPs to move through food chains and the importance of further research to assess their impact [123].

There is a growing concern about the safety of their use in relation to human health [124]. Although AgNPs are used in different fields such as medicine, textiles, or cosmetics, among many others [125], their use for food production can have a greater impact on human health, due to the possible oral exposure to AgNPs (both intentional and accidental), as it is known that nanoparticles can be easily absorbed by the digestive tract and, according to some studies, can even cause inflammation. Even though, after absorption, some NPs are expelled in feces and urine, part of them can pass into the bloodstream and lymphatic system [126]. This is of concern since many studies have indicated that AgNPs are toxic to the mammalian cells that are derived from the skin, the liver, the lung, the brain, the vascular system, and reproductive organs [127]. However (as seen above for plants and crops), the toxicity of AgNPs can be highly dependent on their qualities. For example, the commercial product Argovit^TM^ has been shown to have low cytotoxicity and genotoxicity in tests on mice orally administered AgNPs [128] and low toxicity to human erythrocytes [129] and lymphocytes [130].

## 5. Conclusions

Recent research has shown that AgNPs are a novel tool with great potential in agriculture and sustainable crop production, thanks to their large number of applications both ex vitro and in vitro. Ex vitro, they can be used with different application methods in multiple crops, which makes them a very versatile tool. In in vitro conditions, their addition to the culture medium provides multiple benefits. In both cases, it has been observed that they can improve plant response against stress, promote growth, and even increase the production of bioactive compounds and antioxidants. However, its use under in vitro conditions seems to be much more appropriate, since laboratory conditions can help to obtain more homogeneous results, in addition to avoiding possible adverse effects on the environment. As for ex vitro applications, for example, its use as a foliar spray against viruses, although it has proven to be a suitable alternative, its application in the field may not be optimal, due to its high cost and the uncontrolled release of AgNPs into the environment.

The interaction between nanoparticles and plants is complex. AgNPs can have negative effects on plants or the environment. The difference between an effective treatment with AgNPs and one that produces a toxic effect depends on a number of factors such as the crop, type of application, or the properties of the nanoparticle used (size, concentration, synthesis method, coating, etc.). For this reason, it is necessary to optimize all these variables according to the crop studied; therefore, it is important to conduct further comparative studies that will allow researchers to identify which nanoparticle properties are most important for achieving the desired effect. Future research should also focus on understanding the mechanisms of action of AgNPs, in order to know how they interact with plants and/or pathogens, in addition to focusing on studying the possible long-term negative effects on the environment, animals, and humans, when used in food production. Additionally, there is a need for more field studies to validate the positive results observed in controlled conditions and to assess the performance and safety of AgNPs under real-world agricultural conditions.

The correct implementation of nanotechnology in agriculture has the potential to become a key tool in the future; if applied in a sustainable and efficient manner, it could contribute significantly to ensuring food security in the current context of resource scarcity and climate change.

## Figures and Tables

**Table 1:** Summary of applications of silver nanoparticles against fungal diseases.

Disease	Host Plant	Optimal Dose (mg L^−1^)	Size (nm)	Synthesis Type	Effect	Ref.
Kiwifruit post-harvest rot	*Actinidia* *deliciosa*	75	5	Purchased from NanoComposix	Applied directly to the fruit and reduced the symptoms without leaving Ag residue	[88]
Post-harvest apple fruit brown rot	*Malus domestica* fruits	200	20 ± 5	Green synthesis using crude tea extract	Inhibited the growth of this fungus in vitro and the artificial co-inoculation to the fruit reduced lesions	[89]
Green and blue mold	*Citrus* spp.	-	<100	Purchased from Sigma-Aldrich	Developed a composite of chitosan and AgNPs with good antifungal activity that exceeded chitosan alone	[90]
Rice brown spot	*Oryza sativa*	5 μmol L^−1^	21.2 ± 2.1	Chemical reduction	Foliar treatment reduced the intensity of the disease, increased chlorophyll, and reduced the activity of antioxidant enzymes	[91]
Purple blotch	*Allium cepa*	100	<100	Green synthesis using *Ocimum sanctum* leaf extract	In a field study, an inhibition of 80.93% was obtained with foliar spray applications	[92]

**Table 2:** Summary of foliar applications of silver nanoparticles against plant viruses.

Pathogen	Host Plant	Optimal Dose (mg L^−1^)	Size (nm)	Synthesis Type	Effect	Ref.
ToMV and PVY	*Solanum* *lycopersicum*	50	--	--	Treatments 7 days before infection reduced disease severity	[96]
BYMV	*Vicia faba*,	100	80, 92, and 77	Green synthesis. *Bacillus pumilus*, *B. persicus*, and *B. licheniformi* isolates	Post-infection treatment (24 h after inoculation) prevented destructive symptoms	[97]
		200	8.54	Reduction of silver nitrate	Infection was completely inhibited by 24 h before inoculation treatments	[98]
SHRV	*Cyamopsis* *tetragonoloba*	50	10 to 20.	Green synthesis. Using spore crystal mixture of *Bacillus thuringiensis*	Total suppression of symptoms with treatments at inoculation	[99]
TSWV	*Chenopodium* *amaranticolor*	200	12.6 ± 5	Reduction of silver nitrate	Treatments 24 h after inoculation presented the greatest inhibitory effect	[100]
BBTV	*Musa ×* *paradisiaca*	50	15	Purchased from Sigma company	Post-infection treatment reduced symptoms and increased dry weight and leaf area	[101]
CMV	*Cucurbita pepo*	0.1	36.4 ± 9.3	Green synthesis. Reduction approach using *Ocimum basilicum* extract	Treatment 24 h before or after infection reduced virus and boosted carbohydrates, antioxidants, and resistance gene expression	[102]

Pathogen abbreviations: ToMV: tomato mosaic virus; PVY: potato virus Y; BYMV: Bean Yellow Mosaic Virus; SHRV Sunhemp Rosette Virus; TSWV: tomato spotted wilt virus; BBTV: banana bunchy top virus; and CMV: cucumber mosaic virus.

## Data Availability

Not applicable.
